# Towards Protein Crystallization as a Process Step in Downstream Processing of Therapeutic Antibodies: Screening and Optimization at Microbatch Scale

**DOI:** 10.1371/journal.pone.0025282

**Published:** 2011-09-22

**Authors:** Yuguo Zang, Bernd Kammerer, Maike Eisenkolb, Katrin Lohr, Hans Kiefer

**Affiliations:** 1 Institute of Pharmaceutical Biotechnology, Biberach University of Applied Sciences, Biberach, Germany; 2 Biberach University of Applied Sciences, Biberach, Germany; 3 Boehringer Ingelheim Pharma GmbH&Co. KG., Biberach, Germany; St. Georges University of London, United Kingdom

## Abstract

Crystallization conditions of an intact monoclonal IgG4 (immunoglobulin G, subclass 4) antibody were established in vapor diffusion mode by sparse matrix screening and subsequent optimization. The procedure was transferred to microbatch conditions and a phase diagram was built showing surprisingly low solubility of the antibody at equilibrium. With up-scaling to process scale in mind, purification efficiency of the crystallization step was investigated. Added model protein contaminants were excluded from the crystals to more than 95%. No measurable loss of Fc-binding activity was observed in the crystallized and redissolved antibody. Conditions could be adapted to crystallize the antibody directly from concentrated and diafiltrated cell culture supernatant, showing purification efficiency similar to that of Protein A chromatography. We conclude that crystallization has the potential to be included in downstream processing as a low-cost purification or formulation step.

## Introduction

Therapeutic monoclonal antibodies (mAbs) were introduced into the market in 1986. Since then, processing technologies for this class of therapeutics have seen enormous progress as exemplified by recombinant cell lines producing titers in the range of 10 grams per liter of cell culture. Downstream processing technology currently relies heavily on protein A chromatography, a fast and highly selective capturing step, followed by additional chromatographic procedures such as ion exchange or hydrophobic interaction chromatography. Although the purity of mAb achieved after Protein A chromatography usually exceeds 90%, further purification steps are required to meet the exceptionally high purity targets of biopharmaceuticals. The major drawback of chromatographic procedures is the high cost of adsorption media, which can amount to more than ten thousand US dollar per liter of Protein A resin. Therefore, more economic procedures able to replace at least one chromatographic operation are subject to extensive research.

Protein crystallization, which has been mostly applied in protein structure analysis, has been recognized in principle as a method of protein purification [1], [2]. Within a crystal, protein molecules form a regular lattice able to exclude other proteins as well as misfolded protein molecules of the same type. Therefore, as routinely applied to small molecules, crystallization can also be used as a cheap and scalable purification procedure [3]. Earlier work has demonstrated the feasibility of protein purification by crystallization e.g. for an industrial lipase [4] or the model protein ovalbumin [5]. However, the only biopharmaceutical routinely crystallized at industrial scale and with excellent recovery yields is insulin [6]. Insulin is a small and extraordinarily stable peptide able to refold easily into its native structure even after exposure to organic solvents. It is crystallized late in the purification sequence where most of the impurities have already been removed [4].

Additional benefits of protein crystallization from a formulation perspective are the higher stability of crystalline proteins in comparison to protein solutions, making crystalline formulations an attractive alternative with potentially longer shelf life, and the possibility to control delivery of a protein by making use of crystal dissolution kinetics [7]. The latter has been investigated extensively in the context of insulin formulations [8].

For immunoglobulin, the use of this technique as a means of purification or formulation is not yet a routine procedure. Several authors studied phase behavior of mAbs with the goal to identify a rational approach leading to crystallization conditions [9]–[11]. The work has been complicated by the fact that in addition to crystallization other phenomena such as precipitation, phase separation and the formation of gel-like phases can occur that kinetically trap the system far from equilibrium and as a consequence reduce the yield of crystalline protein or inhibit crystal formation completely.

In our study, we chose an IgG4 mAb that readily crystallizes under a range of conditions, allowing us to optimize the procedure with respect to mass and activity recovery and degree of purity. Focusing on a simple system composed of solvent and crystals, we were able to identify the solubility limit in a phase diagram and use this as the starting point for up-scaling to a process step conforming to GMP requirements. The aim of this work is to show how initial crystallization conditions can be improved and optimized to result in a process step that delivers high purity and high recovery. We want to point out however, that for any individual antibody, those initial conditions have to be identified by screening. There is yet no method available that allows predicting crystallization conditions from protein sequence or general physico-chemical parameters. Nor can crystallization conditions be transferred from one protein to another even if they are very closely related in sequence [12]. The osmotic virial coefficient B_22_, which has been shown to often adopt values within a certain range (“crystallization slot”) under conditions promoting protein crystallization [13], has not proven to become a general predictor for proteins difficult to crystallize [10] [14].

## Materials and Methods

### Antibody

Clarified cell culture supernatant of a CHO derived cell line secreting monoclonal IgG4 type antibody mAb04c as well as Protein A-purified mAb04c were kindly provided by Boehringer Ingelheim Pharma GmbH (Biberach, Germany).

### Crystallization technique

Wizard™ I, II, III Crystal Screen kits were from Emerald BioSystems (Bainbridge Island, US). Basic and Extension Kits were from Sigma (Taufkirchen, Germany).

For protein crystallization, both vapor diffusion and microbatch techniques were utilized. The methods were performed according to Bergfors [15]. 96 well crystallization plates from Corning (Amsterdam, The Netherlands) and Crystalbridge™ (45 µl) from Greiner bio-one (Germany) were used for sitting drops. 24 wells plate (Greiner bio-one) were used for hanging drop and 60 wells plate (Greiner bio-one) for microbatch crystallization. The protein solution was filtered through 0.2 µm filter (Sartorius, Germany) before crystallization.

### Concentration and buffer exchange

Protein A-purified mAb was dialyzed overnight against 20 mM Tris buffer pH 7.0 at 4°C, and was then concentrated to the desired concentration with Vivaspin™ 500 centrifugal filter (30 kDa MWCO, Sartorius) by centrifugation (15000 g, 4°C). Cell culture supernatant was diafiltrated using 7 volumes of 20 mM Tris, 50 mM Histidine, pH 7, and 30 kDa MWCO membrane cassettes (Hydrostat, Sartorius, Germany). Next, mAb was concentrated with Vivaspin™ 500 to the required concentration.

Concentration of purified mAb was determined photometrically at 280 nm using a NanoDrop® 1000 (Thermo Scientific, US) photometer, whereas mAb concentration of culture supernatant was measured by size exclusion HPLC (SE-HPLC) on a Tosoh TSK-GEL 3000 SWXL column at 25°C. HPLC system was HP1100 from Agilent (Waldbronn, Germany). Elution buffer was 0.05 M Tris/0.15 M NaCl, pH 7, the flow rate was 1 ml/min. Chromeleon® software (Dionex, Sunnyvale, US) was applied for chromatogram recording. Protein was detected at 225 nm and integrated elution peaks were compared to a mAb standard calibration curve.

Purity of mAb was estimated by SE-HPLC and by SDS-PAGE on 12.5% Laemmli gels. SDS gels were stained with Coomassie Blue or silver.

### Distinction of crystals and amorphous precipitate

Crystals and amorphous precipitate were distinguished through birefringence using a microscope (Nikon Eclipse 80i, Düsseldorf, Germany) equipped with polarizing filters. Birefringent protein crystals change color upon rotation of the polarizing filter, while amorphous precipitate does not show this behavior.

### Harvest of protein crystal

Crystals were separated from the mother liquor by 10 min centrifugation at 4°C and 10,000 g and washed 3 times with reservoir solution, each volume of the wash reservoir being the same as that of the original sample of crystal suspension. The crystals were redissolved in 100 mM sodium acetate pH 4.0, and stored at 4°C for further analysis.

### Determination of phase diagram

The apparent phase diagram was obtained from microbatch experiments in 60 well plates at room temperature (about 22°C). 1.5 µL of protein solution were mixed with an equivalent volume of crystallization reagent and covered by paraffin oil (Hampton Research, Aliso Viejo, US). Experiments were monitored visually under microscope over a period of five days. No visual changes were detected starting from day three.

In the metastable zone spontaneous nucleation does not occur, and therefore equilibrium cannot be reached through the growth of crystals starting from a supersaturated solution. The solubility limit of mAb04c was therefore measured by dissolving crystals in a protein-free solution until equilibrium reached. MAb04 was first crystallized as controlled by microscopy at 8 mg/ml protein, 8% w/v PEG 8000, 0.2 M Ca(OAc)_2_, 0.1 M Imidazol, pH 7 using microbatch process. Crystals were harvested, homogenized with Seed Bead™ (Hampton Research), and resuspended in deionized water. 20 µl Aliquots of this suspension were added to 1.5 ml centrifuge tubes. Subsequently, 100 µl of precipitant solution as above, but with varying PEG 8000 concentrations was added to each aliquot. The amount of crystallized protein was in excess so that crystals would not dissolve completely. After five days mixing in a Thermomixer Campact (RT, 300 RPM, Eppendorf, Germany), the protein concentration in the supernatant was determined via A_280_ measurement in a NanoDrop photometer following 10 min centrifugation at 10,000 g. Control experiments showed that equilibrium had been attained at this time.

### Binding activity measurement

The functional integrity of the Fc portion of crystallized mAb was confirmed by binding to immobilized Protein A on a Biacore® T100 SPR instrument (GE Healthcare, Germany) as described by the manufacturer.

### Spiking experiments with protein contaminations, DNA and bacteriophage

Host cell proteins (HCPs), virus and DNA are critical impurities in the production of biopharmaceuticals. Therefore, spiking experiments using model protein impurities, genomic DNA and bacteriophage T7 as a virus model were carried out.

For HCP spiking, purified mAb (8.6 mg/ml) was mixed with lysozyme (14.3 kDa, 10 mg/ml, Fluka, Schwitzerland) or BSA (bovine serum albumin, 66 kDa, 10 mg/ml, Carl Roth, Germany) and then crystallized by sitting drop vapor diffusion. Precipitant solution was 10% w/v PEG 8000, 0.2 M Ca(OAc)_2_, 0.1 M Imidazol, pH 7 as above.

For DNA and phage spiking, DNA at a concentration of 25 µg DNA/mg mAb or phage at a concentration of 6.4×10^7^ PFU (Plaque Forming Unit)/mg mAb were added to mAb04c. Crystallization was carried out as above.

Bacteriophage T7 and *E. coli* strain B were kindly provided by A. Kuhn (Hohenheim University, Germany). Bacteriophage concentration was determined by plaque counting following the online protocol [16]. *E. coli* was cultured in LB-Medium pH 7.5. Phage was diluted in 0.85% phosphate buffered saline (PBS buffer: 8 g/l NaCl, 0.2 g/l KCl, 1.44 g/l Na_2_HPO_4_, 0.24 KH_2_PO_4_, pH 7.4). Agar plates contained 20 g pepton, 3.5 g NaCl, 15 g agar, 1.5% w/v glucose, 6.75 mM Na_2_HPO_4_, 8 ml 1% w/v aniline blue (filtered through 0.2 µm sterile filter) per liter. Soft top agar was 7 g agar per liter. All solutions were autoclaved.

DNA concentration was measured using a 96 wells microplate (Greiner bio-one) SYBR® Green I (Invitrogen, Germany) assay [17]. Chromosomal DNA was from salmon sperm (Fluka, Germany). SpectraMax® microplate reader was from Molecular Devices (US). Excitation: 488 nm/Emission: 520 nm.

## Results and Discussion

**Figure 1:**
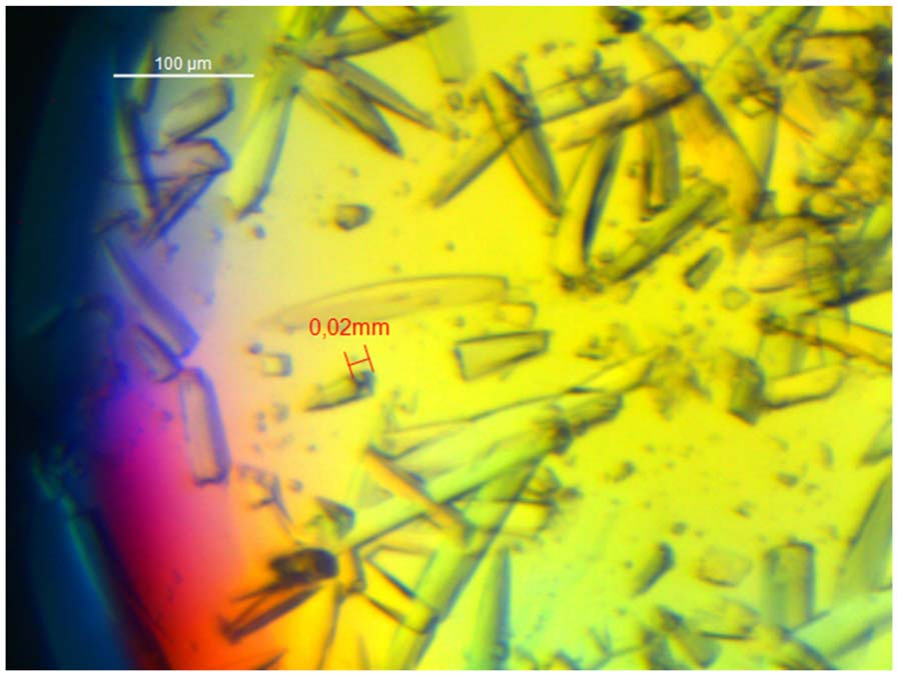
Micrograph of crystals of mAb04c under polarized light. 3 µl microbatch. Conditions: 1.5 µl 20 mg/ml mAb04c in 20 mM Tris, 50 mM Histidine, pH 7 plus 1.5 µl 12% (w/v) PEG 8000, 0.4 M calcium acetate in 0.2 M Imidazol, pH 7, RT. The broken crystal (intentionally) indicates that the dimension of crystal is about 100∼150 µm×10∼20 µm×10∼20 µm.

**Figure 2:**
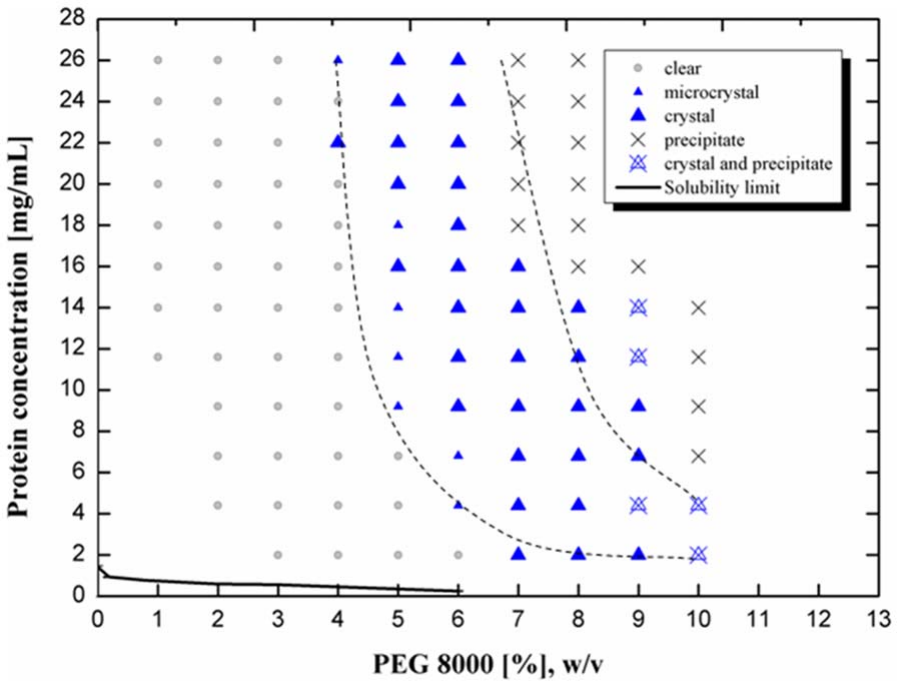
Phase diagram of mAb04c with PEG 8000 as precipitant. Buffer: 0.1 M imidazol, 0.2 M calcium acetate, pH 7.0, RT.

Seven hits (microcrystals or crystals) were found in sparse matrix screening using commercial screen kits. After 3 repetition tests with the 7 conditions found, the most robust condition (10% w/v PEG 8000, 0.2 M Ca(OAc)_2_, 0.1 M Imidazol, pH 7) was chosen as the starting point of a phase diagram. Crystals obtained were coffin-shaped structures (Figure 1). Mechanical rigidity, as qualitatively assessed by micromanipulation using “Crystal tools” (Hampton Research) under microscope, was high compared to “needles” found in other screening experiments. When the seeding stock was prepared (see above) omission of the “Seed bead”, acting as a ball mill, even vigourous vortexing resulted in a preparation that contained relatively large crystals and fragments. We therefore expect that those crystals will not fragment extensively under mechanical stress, e.g. in a stirred crystallizer, and will be suitable for subsequent solid-liquid separation. Protein solubility was studied as a function of the concentrations of protein and PEG 8000 in 0.1 M Imidazol 0.2 M calcium acetate pH 7 at RT (Figure 2). The solubility of mAb decreased with increasing precipitate concentration. Crystals were observed at PEG 8000 concentrations exceeding 5% (w/v). In the protein concentration range between 10 and 14 mg/ml, there was a broad crystallization region between 5 and 9% PEG 8000.

However, this method does not provide the solubility limit, as crystallization does not occur spontaneously in the metastable region where kinetic effects prevent nucleation [12]. Therefore, the solubility limit was determined by preparing saturated solutions in equilibrium with crystalline protein and measuring the protein concentration in the supernatant. The solubility limit (solid line in Figure 2) was found to be located far away from the crystallization zone at protein and precipitant concentrations at least one order of magnitude below the limit of spontaneous crystallization. This result indicates that mAb04c crystallizes spontaneously only at high supersaturation.

**Figure 3:**
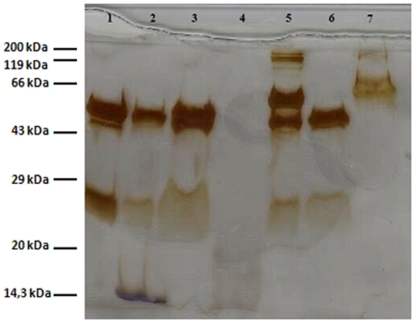
SDS-PAGE of HCP spiking experiment. Silver-staining. Lanes: (1) mAb04c, standard; (2) mother liquor with mAb04c and lysozyme; (3) washed mAb04c crystals from mother liquor with lysozyme; (4) the supernatant from the crystallization with lysozyme; (5) mother liquor with mAb04c and BSA; (6) washed mAb04c crystals from mother liquor with BSA; (7) the supernatant from the crystallization with BSA.

The crystallization of mAb04c in the presence of contaminating protein was examined by spiking with model protein impurities as described by Judge et al [5]. Crystals appeared after 2 days and the crystal shape was indistinguishable from crystals of non-contaminated mAb04c. Crystals were harvested after 5 days and then redissolved in 100 mM Sodium Acetate pH 4. On SDS-PAGE, neither Lysozyme nor BSA still present in the mother liquor was detected in the redissolved mAb crystals (Figure 3, lanes 3 and 6). The only bands detectable resulted from the original mAb protein (IgG light and heavy chains), plus a band probably resulting from partially degraded heavy chain. The contaminants Lysozyme and BSA were only present in the mother liquor (lanes 2 and 5) and in the wash solutions (lanes 4 and 7). Silver staining is reported to detect individual bands exceeding 10 nanograms, indicating that more than 90% of the protein contaminations had been removed.

With the success of the HCP spiking test, we attempted to purify mAb04c from the culture supernatant through one-step crystallization. The supernatant was conditioned and concentrated by diafiltration/ultrafiltration and centrifugation as described above. The volume of crystallization suspension was scaled up from 2 µl to 40 µl in a Crystalbridge™ (20 µl protein solution plus 20 µl reservoir). The reservoir was also scaled correspondingly to 20 ml to keep the ratio constant.

**Figure 4:**
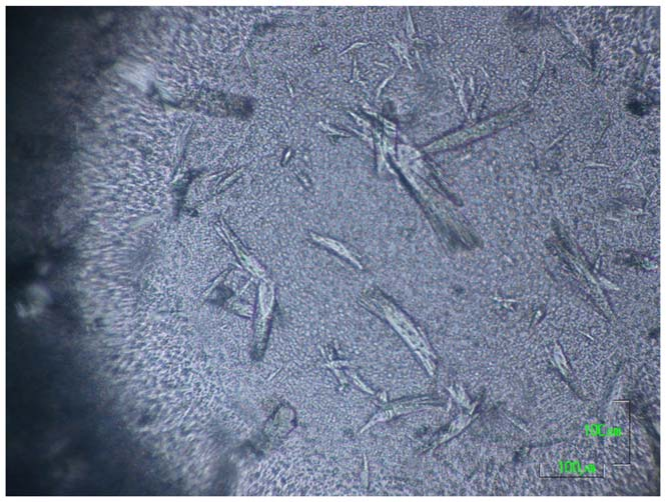
Micrograph of crystalline mAb04c crystallized from clarified culture supernatant. Crystallization condition: sitting drop. Reservoir: 0.1 M imidazol, 0.2 M calcium acetate, 9% w/v PEG 8000. 20 µL clarified culture supernatant (8.3 mg/ml mAb04c) plus 20 µl reservoir, RT. Scale bar 100 µm.

**Figure 5:**
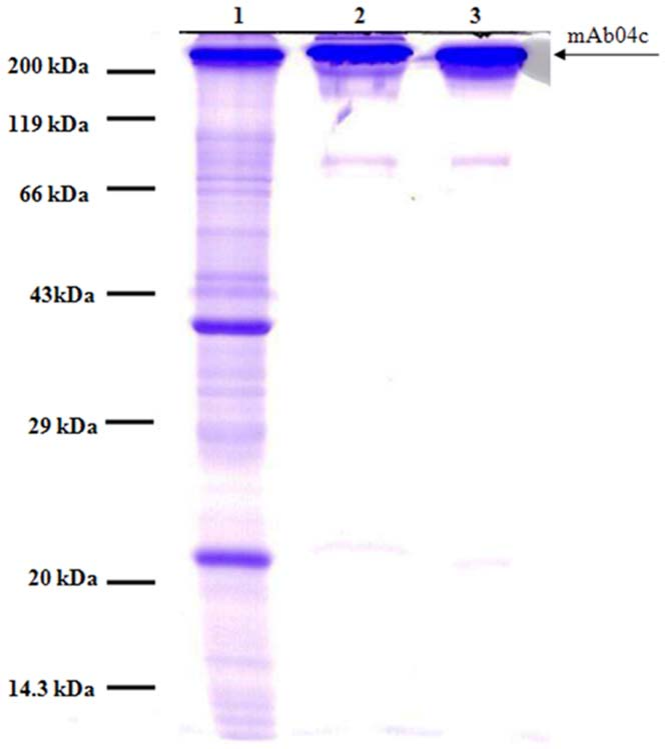
Coomassie blue stained none-reducing SDS-PAGE of mAb04c before and after crystallization or protein A purification. 7 µg of IgG was loaded per lane. Lanes: (1) clarified mAb04c culture supernatant; (2) washed mAb04c crystals, redissolved in 100 mM sodium acetate pH 4.0; (3) mAb04c, purified via protein A chromatography.

**Figure 6:**
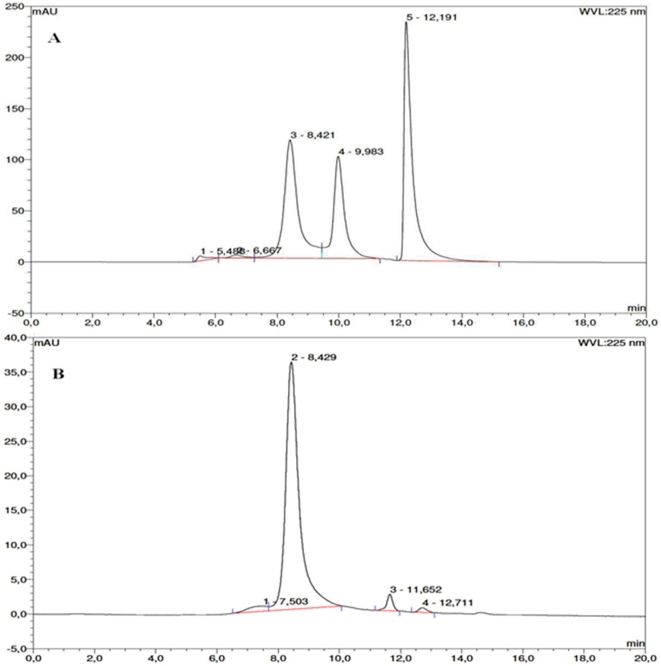
SE-Chromatogram of the sample before and after crystallization. Elution buffer: 0.05 M Tris/0.15 M NaCl, pH 7; flow rate: 1 ml/min; wavelength: 225 nm. (A) Clarified mAb04c culture supernatant. Peak 3: mAb04c, 8.42 min; Peak 4: Contaminating Protein, 9.98 min; Peak 5: Histidine in buffer, 12.19 min. (B) washed mAb04c crystals, redissolved in 100 mM sodium acetate pH 4.0. Peak 2: mAb04c, 8.43 min.

Similar to results of the HCP spiking test, crystals became visible after 2 days (Figure 4). After 5 days the harvested crystals were redissolved in 20 µl 100 mM Sodium Acetate pH 4. On SDS-PAGE using Coomassie staining (Figure 5), the concentration of contaminating proteins was considerably reduced (lane 2). A sample of IgG purified by protein A chromatography is shown as a benchmark reference (lane 3). The purity and amount of mAb in each fraction was then accessed by HPLC-SEC (Table 1). Before crystallization, the purity of mAb04c in solution (8.3 mg/ml in 20 µl supernatant) was 42% of total protein according to peak integration. The analysis of redissolved crystals (2.6 mg/ml mAb in 20 µl buffer) showed that no significant oligomer arose from crystallization (Figure 6). The purity was increased significantly to 90% of total protein. Still, only 31.3% of the mAb originally present in the culture supernatant was recovered in crystals, while the larger portion (67.5%) was still found in the crystallization supernatant. In comparison, the yield of crystalline mAb04c that had undergone a prior protein A purification was 95% with no detectable product in the mother liquor.

**Table 1:** Purity and Recovery of crystallized mAb04c.

Fraction	Amount of mAb04c (µg)	Recovery (%)	Purity (%, estimated)
Clarified culture supernatant	166	100	42
washed mAb 04c crystals*	52	31.3	90
supernatant from the crystallization	112	67.5	10

*Protein redissolved in 100 mM sodium acetate pH 4.0.

The binding ability of mAb04c to protein A before and after crystallization was determined via Biacore®. The association rate constants (k_a_) were compared to evaluate a potential affinity loss. Before crystallization, k_a_ was determined to 3.35×10^6^ L/(mol*s), while after crystallization k_a_ was 4.81×10^6^ L/(mol*s). Apparently, crystallization had no influence on the affinity of the Fc-Region for protein A.

In spiking experiments, T7 bacteriophage and chromosomal DNA were added to the mAb solution before crystallization in order to challenge the ability of the crystallization process to remove non-protein impurities. Results are summarized in Table 2. LRV (Log reduction value) was 1.4 for DNA and 2.2 for T7 phage.

**Table 2:** Results of DNA and phage spiking.

	Spiking concentration	Concentration in re-dissolved crystals	LRV
T7 phage [PFU/mg mAb]	6.4×10^7^	4.5×10^5^	2.2
DNA [µg/mg mAb]	25	1.1	1.4

In the present work, we examined the possibility of establishing crystallization as a process step for purification and formulation of a monoclonal antibody. High yields were achieved when crystallization was introduced after chromatographic purification, but not when conditioned cell culture supernatant was used as starting material. Interestingly, crystallization from culture supernatant was nevertheless possible and resulted in efficient removal of contaminants.

**Figure 7:**
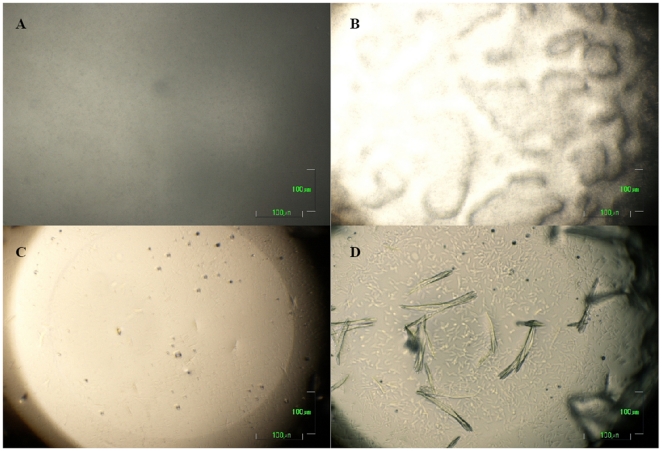
Time course of microbatch crystallization. Protein: 8 mg/ml mAb04c, crystallization buffer: 8% (w/v) PEG 8000, 0.2 M calcium acetate 0.1 M imidazol, pH 7.0, RT. Time after mixing: (A) 0 hrs, (B) 2 hrs, (C) 10 hrs, (D) 70 hrs. Scale bar 100 µm.

We frequently observed that within the crystallization region (area labeled by triangles in Figure 2), protein initially precipitated directly after mixing (Figure 7a), while crystals were detectable under the microscope only after a 3–4 hour lag period (Figure 7b & 7c). This effect is known from literature. Even when the crystalline form of protein is more stable than its amorphous form, slow nucleation can delay the formation of crystals with respect to amorphous precipitate [18], [19]. Precipitate concentration will then decrease at the same time as crystals grow. Two models have been proposed to explain the growth of crystals from precipitates: phase transition and Oswald ripening [20]. Phase transition is a process where crystals form at the expense of a solid amorphous phase, whereas Oswald ripening describes growth of a few crystals at the expense of many microcrystals [21]. Ng et al. [20] reported that both processes can occur simultaneously. Unfortunately, the birefringence method described above is not applicable to microcrystals smaller than the microscope resolution limit. In lack of a method distinguishing between amorphous precipitate and microcrystals, we could not decide which of the above processes was dominant.

To our knowledge, this is the first determination of the solubility limit at equilibrium for an IgG protein. Ahamed et al. [9] describe an apparent solubility limit in a system where precipitate, but not crystals formed. In comparison to their findings, we found a much lower solubility limit. This could of course be attributed to using a different protein, but Ahamed et al. point out that they would expect a lower solubility limit at equilibrium, i.e. in the presence of crystals as opposed to precipitate. The same antibody was studied by Lewus et al. [10] recently, and crystallization conditions were now identified at a different pH. However, here the solubility limit was not determined unequivocally as concentrations measured in the supernatant decreased over the course of 2 months (Fig. 4 of above reference). The large gap found in our experiments between equilibrium solubility and zone of spontaneous crystallization appears unexpected at first sight, but might be a feature of IgGs in general. However, similar studies on other model antibody systems will have to be performed before this view can be corroborated. From a practical point of view, our result means that in a continuous process using heterogeneous nucleation, protein should be crystallizable at much lower protein concentration such as 2 mg/ml and at precipitant concentrations of 7% PEG 8000 or even lower.

Removal of protein contaminations and virus was successful and comparable to Protein A chromatography. DNA removal however was marginal. This may be due to adsorption of DNA to the crystal surface by electrostatic interaction (mAb04c is positively charged at the pH of crystallization). We found that reducing the spiking concentration to 2.5 µg DNA/mg mAb, resulted in 0.35 µg DNA/mg mAb (data not shown), i.e. in an even lower LRV.

Recovery yield of crystallized mAb that had undergone prior protein A purification reached 95%. This is expected when crystallization conditions are far above the solubility limit as revealed by the phase diagram. However, such high yields could not be achieved when cell culture supernatant was subjected to crystallization. Here the recovery of ∼30% indicated that either solubility of mAb had dramatically increased due to other compounds present in the culture supernatant, or crystallization had simply slowed-down. Microscopic examination indicated that onset of crystal formation was significantly delayed when culture supernatant was used instead of purified antibody: in the latter case, crystals could already be detected after 10 hrs, whereas the same process took 48 hrs starting from culture supernatant. Still, crystal shapes were similar in both cases.

Significant nucleation is the primary requirement for crystal formation. The nucleation rate depends in principle on the solubility of the protein, the degree of supersaturation and the interfacial free energy (γ) between solute and solution [22]. The first two contributions are not expected to be significantly influenced by the presence of impurities. As for the interfacial free energy, from thermodynamic aspect, an increase of the nucleation rate should be observed when impurities are present in the solution [22], because any adsorption onto the nucleus decreases γ [23]. However, kinetically, adsorbing impurities can keep the nucleus at a subcritical size [22]. The nucleation rate hence can be drastically reduced. It was reported that impurities present at 10^−5^ mol/L can decrease the nucleation rate by ten orders of magnitude [24]. Biostelle et al. [22] point out that there is a competition between the adsorption kinetics of the impurities and the adsorption and integration kinetics of the solute, hence, a longer nucleation time is needed.

Moreover, this competition may further affect the growth of crystals when nuclei develop and begin to transform into crystals. Impurities can adsorb to the surface of the growing crystal, inhibit the addition of free molecules and thereby decrease the growth rate. Because of the negative effect of impurities in both stages of crystallization, the observed delay in mAb04c crystal formation in the presence of contaminating protein was not unexpected. In our work we did not study which stage was prone to be affected by the presence of impurities.

A low recovery rate (31.3%) was observed, when mAb04c was crystallized from culture supernatant. In a future scaled-up process, nucleation and crystallization rates will have to be controlled e.g. by seeding, feeding protein during crystallization or evaporating solvent. Therefore the yield currently obtained should be subject to significant improvement when working at larger scale.

### Conclusion

Protein crystallization has the potential to be introduced as a purification step in downstream processing of mAbs, although our present results so far show that it will likely be more useful in a later purification step and not in initial capturing of the product. Here we demonstrate purification of a monoclonal antibody in a single crystallization step from clarified cell culture supernatant to >90% purity, though with yet unsatisfactory yield. Aggregate formation was negligible as shown by SE-HPLC and binding activity to Protein A was not affected by crystallization. The current crystallization time of several days required to reach equilibrium is partly due to the shortcomings of vapor diffusion and should be subject to considerably improvement in a seeded batch process. Current work is focused on up-scaling and optimization of the process with respect to higher yields. We are well aware that at present only a handful of intact IgGs have been successfully crystallized and that there is no generic method available to identify crystallization conditions rapidly for new antibodies. However, the recent results of Lewus et al. [10], who were able to crystallize an antibody where previously no crystallization conditions have been found [9], are encouraging. More work on a larger set of target mAbs will have to be done before the usefulness of this method can be evaluated in a broader context.
